# S1 Employs Feature-Dependent Differential Selectivity of Single Cells and Distributed Patterns of Populations to Encode Mechanosensations

**DOI:** 10.3389/fncel.2019.00132

**Published:** 2019-04-05

**Authors:** Yoo Rim Kim, Chang-Eop Kim, Heera Yoon, Sun Kwang Kim, Sang Jeong Kim

**Affiliations:** ^1^Department of Physiology, Seoul National University College of Medicine, Seoul, South Korea; ^2^Department of Biomedical Sciences, Seoul National University College of Medicine, Seoul, South Korea; ^3^Department of Physiology, College of Korean Medicine, Gachon University, Gyeonggi-do, South Korea; ^4^Department of Science in Korean Medicine, Graduate School, Kyung Hee University, Seoul, South Korea; ^5^Department of Physiology, College of Korean Medicine, Kyung Hee University, Seoul, South Korea; ^6^Neuroscience Research Institute, Seoul National University College of Medicine, Seoul, South Korea

**Keywords:** brushing, pinch, stimulus feature, primary somatosensory cortex, two-photon Ca^2+^ imaging

## Abstract

The primary somatosensory (S1) cortex plays an important role in the perception and discrimination of touch and pain mechanosensations. Conventionally, neurons in the somatosensory system including S1 cortex have been classified into low/high threshold (HT; non-nociceptive/nociceptive) or wide dynamic range (WDR; convergent) neurons by their electrophysiological responses to innocuous brush-stroke and noxious forceps-pinch stimuli. Besides this “noxiousness” (innocuous/noxious) feature, each stimulus also includes other stimulus features: “texture” (brush hairs/forceps-steel arm), “dynamics” (dynamic stroke/static press) and “intensity” (weak/strong). However, it remains unknown how S1 neurons inclusively process such diverse features of brushing and pinch at the single-cell and population levels. Using *in vivo* two-photon Ca^2+^ imaging in the layer 2/3 neurons of the mouse S1 cortex, we identified clearly separated response patterns of the S1 neural population with distinct tuning properties of individual cells to texture, dynamics and noxiousness features of cutaneous mechanical stimuli. Among cells other than broadly tuned neurons, the majority of the cells showed a highly selective response to the difference in texture, but low selectivity to the difference in dynamics or noxiousness. Between the two low selectivity features, the difference in dynamics was slightly more specific, yet both could be decoded using the response patterns of neural populations. In addition, more neurons are recruited and stronger Ca^2+^ responses are evoked as the intensity of forceps-pinch is gradually increased. Our results suggest that S1 neurons encode various features of mechanosensations with feature-dependent differential selectivity of single cells and distributed response patterns of populations. Moreover, we raise a caution about describing neurons by a single stimulus feature ignoring other aspects of the sensory stimuli.

## Introduction

It is well known that the primary somatosensory (S1) cortex plays an important role in the perception and discrimination of the mechanosensations. The S1 cortex receives innocuous and noxious somatosensory inputs from the thalamus, and is involved in sensory-discriminative aspects of pain including location, duration, and intensity (Bushnell et al., 1999; Apkarian et al., 2005; Basbaum et al., 2009). So far, electrophysiological studies investigating the role of S1 cortex for touch and pain have often focused on the responses of single neurons (Matsumoto et al., 1987; Quiton et al., 2010; Whitsel et al., 2010), or the population response for stimuli with a single feature (Reed et al., 2008; Lefort et al., 2009), limiting the opportunity of understanding the population-level encoding strategy of S1 cortex for multiple features. Hence, the unexplored question is how multiple S1 neurons simultaneously encode diverse features of touch and pain sensation, such as noxiousness, texture, or dynamics.

Traditionally, the somatosensory neurons in the central nervous system (CNS) have been classified as low threshold (LT), high threshold (HT) or wide dynamic range (WDR) neurons according to their electrophysiological responses to innocuous and noxious stimuli. For instance, neurons that respond best to brush-stroke are classified as LT; neurons only responsive to pinching with forceps are classified as HT; those responding to both brush and pinch but more intensely to pinch stimulus are classified as WDR (Lamour et al., 1983; Chung et al., 1986; Senapati et al., 2005). Despite the widespread adoption of this approach to identify the characteristics of the neurons in terms of the noxiousness (innocuous/noxious) or intensity (weak/strong) feature, however, it should be recognized that those stimuli can be qualitatively different (Chung et al., 1986). They are not only characterized by features such as noxiousness and intensity, but also by texture (brush hairs/forceps steel arm) and dynamics (dynamic stroke/static press), even though simple interpretations such as LT or HT have been made in many previous studies. In particular, this consideration will be more important if the neurons of interest can process multiple features of information. S1 neurons seem to be able to encode diverse features of sensory information compared to neurons in the spinal cord (Carter et al., 2014; Saal and Bensmaia, 2014), where the concept of LT/HT/WDR was originally proposed.

Here, we used *in vivo* two-photon Ca^2+^ imaging to simultaneously record the activity of layer 2/3 neurons in the S1 cortex in lightly anesthetized mice in response to cutaneous stimuli using brush and forceps with diverse features such as noxiousness (innocuous/noxious), intensity (weak/strong), texture (brush hair/forceps steel arm), and dynamics (dynamic stroke/static press). We identified individual neurons with distinct tuning properties to texture, dynamics and noxiousness features of the cutaneous stimuli, as well as many broadly tuned neurons. Overall, the majority of the tuned neurons showed a highly selective response to the difference in texture, but low selectivity to the difference in dynamics or noxiousness. Both dynamics and noxiousness features could be decoded using the response patterns of neural populations, implying all the relevant information of these features is being processed in a distributed manner in the S1 cortex. Our findings show how the neural population in S1 encode sensory information with multiple features and also suggest that the tuning property of S1 neurons does not match with the previous concept of LT/HT/WDR.

## Materials and Methods

### Animal Preparation and Virus Injection

All experimental procedures were approved by the Seoul National University Institutional Animal Care and Use Committee and performed in accordance with the guidelines of the National Institutes of Health. We used C57BL/6 male mice (5–6 weeks old at the surgery). All surgeries were conducted under isoflurane anesthesia (1%–1.5%). A cranial window was made over the left S1 cortex hind paw area (size, 2 × 2 mm; center relative to Bregma: lateral, 1.5; posterior, 0.5 mm; Eto et al., 2011; Kim and Nabekura, 2011). The animal skull was opened above the S1 cortex and a small craniotomy was carefully performed using a #11 surgical blade (Jin et al., 2016). The dura was left intact. This exposed cortex was superfused with ACSF. And we injected adeno-associated virus expressing GCaMP6s (AV-1-PV2824; produced by University of Pennsylvania Gene Therapy Program Vector Core) into the S1 cortex at 2–4 sites (30–50 nl per site; 200–300 μm from the surface) using a broken glass electrode (20–40 μm tip diameter). Finally, the exposed cortex was covered with a thin cover glass (Matsunami, Japan) and the margin between the skull and the cover glass was tightly sealed with Vetbond (3M). Mouse body temperature was maintained between 36 and 38°C using a heating pad (IL-H-80, Live Cell Instrument) during animal surgery and imaging experiments. Dexamethasone (0.2 mg/kg) and meloxicam (20 mg/kg) were administered by subcutaneous injection prior to surgery to minimize the potential edema and inflammation (Otazu et al., 2015; Jin et al., 2016). Imaging sessions started 2 weeks after the surgery. Only two mice were housed in each cage in the vivarium to minimize stress on each other. The vivarium was controlled with 12 h light/dark cycle and all experiments were performed during the daylight hours.

### Peripheral Stimulation During Imaging Experiments

All stimuli were delivered to the right hind paw using brush or stainless forceps. For texture and dynamics features experiment (*N* = 4 mice, Figure 2), brush and forceps stimuli were subdivided into Brush-stroke (B-stroke, 1-Hz stroke by brush), Brush-press (B-press, light press by brush), Forceps-stroke (F-stroke, stroke by forceps) and Forceps-press (F-press, <2 g light press by forceps) according to their texture and dynamics (Tables s 1, 2). Stimuli were applied for 5 s per stimulus and inter-stimulus intervals were 15–20 s to avoid sensitization. For aversive noxiousness and intensity experiment (*N* = 6 mice, Figure 3), pinch stimuli were delivered by the experimenter using a rodent pincher meter [Rodent pincher, BIOSEB] for 3 s per stimulus to minimize sensitization (F-pinch; Poisbeau et al., 2005). Inter-stimulus intervals were 20 s and stimulation intensities were P0 < 2 g, P1 = 100 g, P2 = 200 g and P3 = 300 g. The intensities were manually controlled by the experimenter (Kim Y. S. et al., 2016).

**Figure 1:**
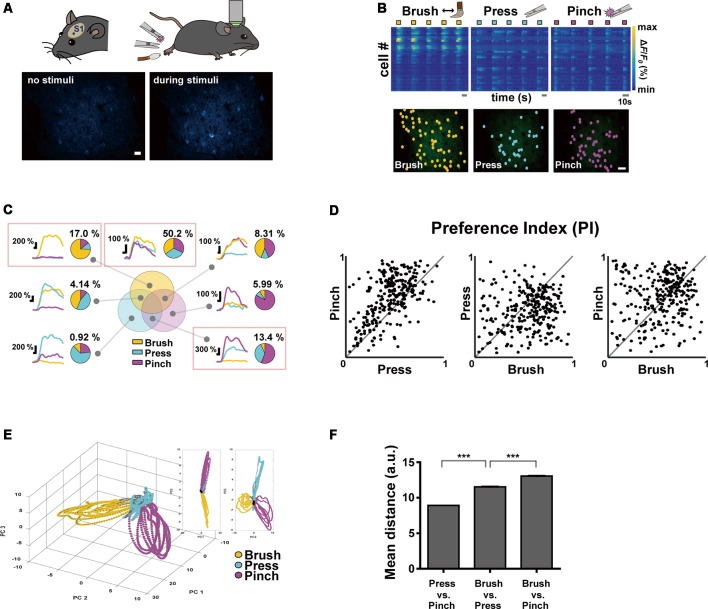
Neural response properties evoked by innocuous and noxious stimuli in the mouse primary somatosensory (S1) cortex. (**A**; Top) Schematic diagrams of experimental approach: a craniotomy was made over the S1 cortex corresponding to the hind limb in the left hemisphere and three types of sensory stimuli were delivered to the right hind paw of anesthetized head-fixed mice using brush and forceps. (Bottom) Representative *in vivo* two-photon Ca^2+^ fluorescence images of layer 2/3 S1 neurons during rest and pinch stimulation with forceps. Scale bar, 20 μm. (**B**; Top) Color-coded raster plots of representative Ca^2+^ transients in S1 neurons in response to Brush, Press and Pinch. Each stimulus was applied in five trials for 5 s. Color-coded *ΔF*/*F*_0_ (%) ranges from 0 to 1,000. Scale bar, 10 s. (Bottom) Spatial distribution of responsive neurons to Brush (yellow), Press (cyan) or Pinch (purple) for an example mouse. Scale bar, 50 μm. **(C)** Seven types of Ca^2+^ responses of the neurons responding to three different stimuli: on the right side of each response type, a representative pie chart shows proportions of the neurons responding to Brush (yellow), Press (cyan) and Pinch (purple), and their percentage to the total. Each portion of the Venn diagram corresponds to a type of neuron. Three red-boxed figures point the proportions of Brush specific (17.0%), Press/Pinch preferred (13.4%) and broadly tuned (50.2%) neurons (*n* = 217 cells from four mice). **(D)** Scatter plots of the preference indexes (PIs) of individual neurons for two different stimuli: (Left) Press vs. Pinch; (Middle) Brush vs. Press; (Right) Brush vs. Pinch (*n* = 217 cells from four mice). **(E)** An example of state-space representation of population activity patterns in response to the three stimuli from an example mouse. N-dimensional activity patterns (N, number of cells) over time were projected onto their two or three principal components *via* dimensionality reduction method. Each color (yellow, cyan, and purple) corresponds to each type of the stimuli. Black dots indicate states before stimuli onset and gray dots indicate states of inter-stimuli time. **(F)** Mean Euclidean distances between states in the state-space represented in **(E)**. Distances were calculated between states in Press vs. Pinch (8.92 ± 0.01, 46,872 pairs from four mice), Brush vs. Press (11.54 ± 0.02, 47,524 pairs from four mice) and Brush vs. Pinch (13.05 ± 0.03, 47,304 pairs from four mice). Data are represented as mean ± s.e.m. Statistics was performed with one-way ANOVA with Tukey’s *post hoc* test, *F* = 6,577, ***p < 0.001.

**Figure 2:**
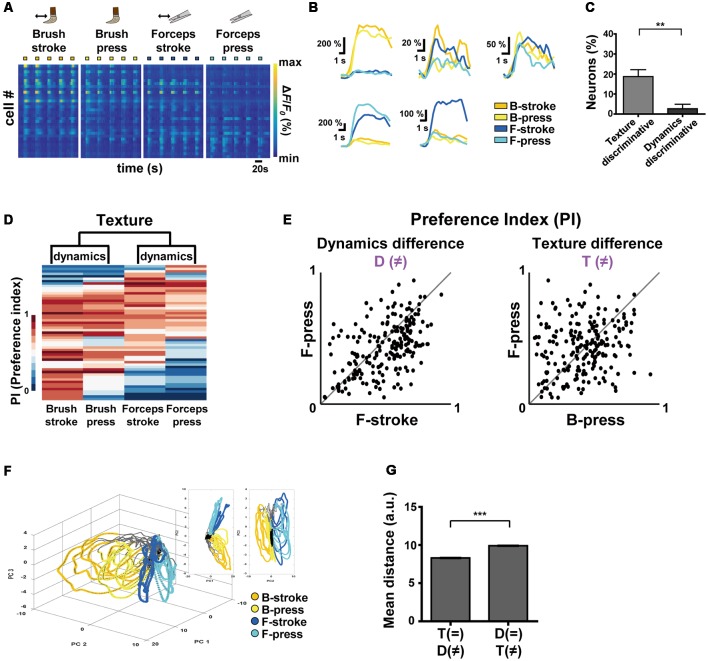
Differential encoding of texture and dynamics features of innocuous stimuli by S1 neurons. **(A)** Color-coded raster plots of representative Ca^2+^ transients in S1 neurons in response to Brush-stroke (B-stroke), Brush-press (B-press), Forceps-stroke (F-stroke) and Forceps-press (F-press). Each stimulus was applied in five trials for 5 s. Color-coded *ΔF*/*F*_0_ (%) ranges from 0 to 800. Time scale, 20 s. **(B)** Examples of various Ca^2+^ responses from five neurons to B-stroke (yellow), B-press (light-yellow), F-stroke (blue) and F-press (cyan) stimuli. **(C)** The percentage of texture-discriminative neurons (preferentially responsive to B-stroke/B-press or F-stroke/F-press, 18.75% ± 3.43%) and that of dynamics-discriminative (preferentially responsive to B-stroke/F-stroke or B-press/F-press, 2.67% ± 2.21%) neurons. Data are represented as mean ± s.e.m. Statistics was performed with a two-tailed unpaired *t*-test (*n* = 208 cells from four mice; *t* = 3.94; **p = 0.0015). **(D)** Hierarchical clustering analysis based on Ca^2+^ responses of S1 neurons to the different textures or dynamics. Ca^2+^ responses of each cell were normalized to a single PI per each stimulus and four types of stimuli were clustered according to the PIs of cells. **(E)** Scatter plots of PIs of individual neurons for two different stimuli: (Left) Dynamic difference, F-stroke vs. F-press; (Right) Texture difference, B-press vs. F-press (*n* = 208 cells from four mice). **(F)** An example of State-space representation of population activity patterns in response to the four stimuli. N-dimensional activity patterns (N, number of cells) over time were projected onto their two or three principal components *via* dimensionality reduction method. Each color (yellow, light-yellow, blue and cyan) corresponds to each type of the stimuli. Black dots indicate states before stimuli onset and gray dots indicate states of inter-stimuli time. **(G)** Mean Euclidean distances between states in the state-space represented in **(F)**. Distances were calculated between states that differ in dynamics (F-stroke vs. F-press and B-stroke vs. B-press, 8.308 ± 0.014), and texture (B-stroke vs. F-stroke and B-press vs. F-press, 9.910 ± 0.014). Data are represented as mean ± s.e.m (97,886 pairs from four mice). Statistics was performed with a two-tailed unpaired *t*-test (*t* = 81.71; **p < 0.01, ***p < 0.0001).

**Table 1:** Explanatory table for the different types of stimuli applied to the experiment in each figure using a brush or forceps.

	Figure 1	Figure 2			Figure 3	
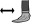	Brush	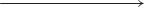	B-stroke			
			B-press			
			F-stroke			
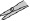	Press	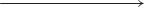	F-press				
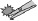	Pinch	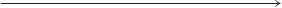			F-pinch	P0
						P1
						P2
						P3

**Figure 3:**
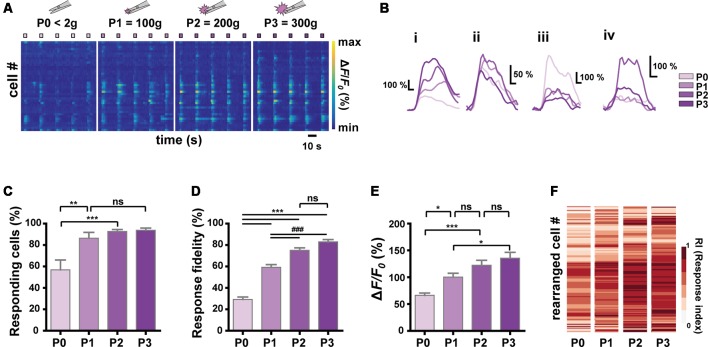
Relationship between stimulus intensity and Ca^2+^ responses of S1 neurons. **(A)** Color-coded raster plots of representative Ca^2+^ transients in S1 neurons in response to the four different intensities (P0 < 2 g, P1 = 100 ± 30 g, P2 = 200 ± 30 g and P3 = 300 ± 30 g pressure). Each type of stimuli was applied in five trials for 3 s. Color-coded *ΔF*/*F*_0_ (%) ranges from 0 to 1,000. Time scale, 10 s. **(B)** Examples of various Ca^2+^ responses from four neurons to the graded pinch stimuli. Time scale, 1 s. **(C)** The relationship between the number of responding cells and the stimulus intensity (*N* = 6 mice; one-way ANOVA, *F* = 10.16; *p* = 0.0003). **(D)** The relationship between the response fidelity of neurons and the stimulus intensity (*n* = 197 cells from six mice; one-way ANOVA, *F* = 104; *p* < 0.0001). **(E)** The relationship between Ca^2+^ transients amplitude and the stimulus intensity (*n* = 197 cells from six mice; one-way ANOVA, *F* = 13.54; *p* < 0.0001). Data are represented as mean ± s.e.m. One-way ANOVA test was performed with Tukey’s *post hoc* for multiple comparisons. ns *p* > 0.05, *p < 0.05, **p < 0.01, *** and ^###^*p* < 0.001. **(F)** Heat maps from the response indexes (RIs) of cells to the stimuli with different intensities (P0, P1, P2, and P3). Cells (rows) are rearranged for the purpose of visualization (*n* = 197 cells from *N* = 6 mice).

*Innocuous Brush, Innocuous Press and noxious Pinch stimulus are applied in Figure 1. B-stroke and F-press are relabeled terms of Brush and Press stimulus in Figure 1, respectively, and in addition, B-press and F-stroke are added in Figure 2. The Pinch stimulus in Figure 1 is applied in four different intensities in Figure 3*.

**Table 2:** Explanatory table for the different types of stimuli applied to the experiment in Figure 2 and Figure 3.

			Texture	Noxiousness	Dynamics
Figure 2	B-stroke		Brush	Innocuous	Dynamic
	B-press			Innocuous	Static
	F-stroke		Forceps	Innocuous	Dynamic
	F-press			Innocuous	Static
Figure 3	F-pinch	P0	Forceps	Innocuous	Static
		P1			
		P2		Noxious	
		P3

*Each stimulus was classified by texture, noxiousness and dynamics using a brush or forceps. F-pinch stimulus was subdivided into four intensities. P0 < 2 g, P1 = 100 g, P2 = 200 g and P3 = 300 g*.

### *In vivo* Two-Photon Calcium Imaging of Layer 2/3 Neurons

Calcium imaging was performed with a two-photon microscope (Zeiss LSM 7 MP, Carl Zeiss, Jena, Germany) equipped with a water immersion objective (Apochromat 20×, NA = 1.0, Carl Zeiss). Two-photon excitation for GCaMP6s imaging (900 nm) was provided by a mode-locked Ti: sapphire laser system (Chameleon, Coherent). Imaging was acquired using ZEN software (Zeiss Efficient Navigation, Carl Zeiss). All the experiments were conducted under anesthesia with isoflurane (1%) and the body temperatures of mice were maintained between 36 and 38°C using a heating pad (IL-H-80, Live Cell Instrument). For layer 2/3 neurons calcium imaging, time-lapse imaging (512 × 300 pixels, 0.4 μm/pixel, two line steps, 0.229 s per frame) was performed with imaging depth of 180–220 μm from the surface.

### Data Analysis

We manually selected regions of interests (ROIs) corresponding to individual neurons by circling each fluorescence, using time-lapse movie program. Customized scripts in MATLAB were used to analyze the calcium transient signals. Calcium signal amplitudes were calculated as *ΔF*/*F*_0_ (*ΔF* = *F* − *F*_0_) for each cell. *F*_0_ means the baseline fluorescence signal calculated by averaging lowest 30% of all fluorescence signals from individual traces. Responding neurons were defined as neurons with fluorescence change >30% of *F*_0_, and we further analyzed only responding neurons. To determine the tuning properties of each cell for each stimulus, we defined and computed preference index (PI) that ranges from 0 to 1. Preference index of cell *i* for stimulus *j (PI_ij_)* was defined as:

$$PI_{ij}=\frac{\bar{P_{ij}}}{{\text{Max}}_{i}}$$

where $\bar{P_{ij}}$ is the mean of the peak values of cell *i* for stimulus *j* across repeated trials (*P_ijk_*) and *P_ijk_* was determined as the highest value of amplitude during each trial *k* for stimulus *j* in cell *i*. Max_i_ is the highest value that cell *i* showed during the experiments. We defined cell *i* to be “preferentially responsive” or “tuned” to stimulus *j* when *PI_ij_* is larger than 0.8*$\bar{PI_{i}}$, where $\bar{P_{ij}}$ is the average of *PI_ij_* for all the given stimulus. Response index (RI) was defined the same as PI except that RI is computed for one kind of stimulus (noxious) with different intensities rather than different kind of stimuli. To represent population activity patterns of S1 neurons to different stimuli in the low dimensional space, principal component analysis (PCA), a dimensionality reduction method, was used. N-dimensional activity patterns (*n*, number of cells) over time were projected onto their two or three principal component axes (each axis being a linear combination of *n* neural activities). In order to understand the encoding strategy of S1 neurons for each stimulus, we constructed scatter-plots of PI values (PI scatter plots) between each pair of two stimuli. Then, the Euclidean distances were computed and averaged between each scatter-plotted point and “equally tuned” line which passes through points of “stimulus 1 = stimulus 2.” To standardize the average distance for each pair of stimuli, 100,000,000 reshuffled pairs of PIs were constructed for each pair of stimuli. The reshuffled pairs of PIs conserve the original PI values for each cell, but no associations between two PIs remain. Means and standard deviations of distances were computed from these permutation data and z-distances were calculated using the means and standard deviations. To test the significance of z-distances (i.e., whether there is any association between each pair of PIs in cells), empirical *p*-values were directly computed from the permutation sets and Bonferroni corrections were conducted. To investigate whether the sensory information of the stimulus with each feature is encoded in S1 as a pattern of the population activity, we applied the supervised machine learning algorithm, *k-nearest* neighbor classifier (*k* = 5, and Euclidian metric). Vectors *P_ijk_* containing (*i* = 1, …, *n*; *n* = 101 cells from six mice) were used as training and test samples for stimulus *j*. Ten-fold cross-validation was used to evaluate the decoding performance. This validation procedure ensures trained classifiers to be tested using data unseen during training phases. Empirical *p*-values were computed with 100,000,000 random permutations of the label (features to predict).

### Statistics

**Figure 4:**
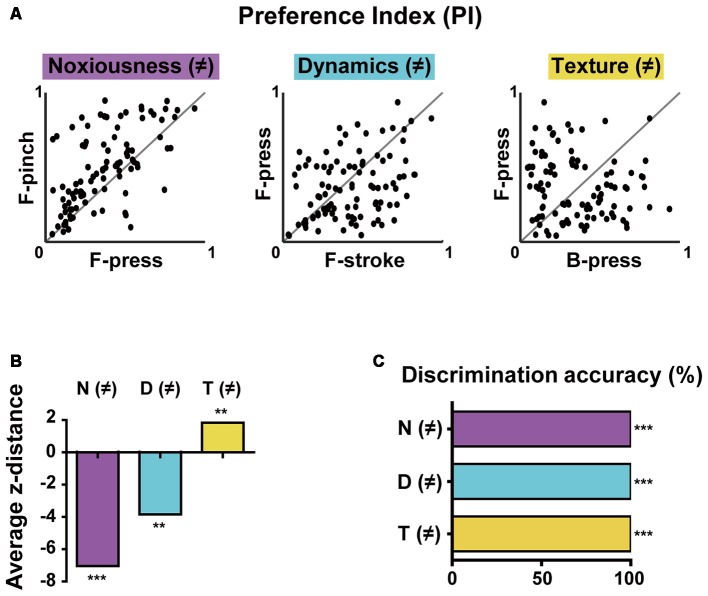
Differential selectivity to multiple stimulus features and decoding features of the population activity in the S1 cortex. **(A)** PI scatter plots of non-broadly tuned neurons between two stimuli that differ in noxiousness (F-press vs. F-pinch), dynamics (F-stroke vs. F-press), or texture (B-press vs. F-press; *n* = 101 cells from six mice). Noxiousness (M)-purple, Dynamics (D)–cyan and Texture (T)–yellow. **(B)** Average z-distances between PIs (i.e., tuning property) and “equally tuned” lines for pairs of stimuli. Empirical *p*-values were calculated by permutation tests (average z-distance = −7.076, ***p < 0.001 for F-press and F-pinch, average z-distance = −3.905, **p < 0.01 for F-stroke and F-press, average z-distance = 1.885, **p < 0.01 for B-press and F-press; Bonferroni-corrected). **(C)** Decoding performance for stimuli that differ in noxiousness (F-press vs. F-pinch), dynamics (F-stroke vs. F-press), or texture (B-press vs. F-press) by neural population activity (***p < 0.001).

Data were processed, analyzed and plotted using custom-written MATLAB scripts (MathWorks, Natick, MA, USA) or Prism software (Graph Pad Software, San Diego, CA, USA). All data are represented as mean ± s.e.m. Two-tailed unpaired *t*-test (Figures 2C,G), Wilcoxon signed-rank test (Supplementary Figure S1C), two-tailed paired *t*-test (Supplementary Figure S1D), one-way ANOVA with Tukey’s *post hoc* test (Figures 1F, 3C–E), and permutation tests with Bonferroni-corrections (Figures 4B,C) were used to determine the significance in statistical comparisons. The differences were considered significant if a *p* value is below 0.05. NS indicates *p* > 0.05, *indicates *p* < 0.05, **indicates *p* < 0.01, *** and ^###^ indicates *p* < 0.001.

## Results

### Neural Response Patterns to Innocuous and Noxious Stimuli in the S1 Cortex of Mouse

Using two-photon Ca^2+^ imaging in lightly anesthetized mice expressing GCaMP6s in the layer 2/3 neurons of the left S1 cortex, we first tried to determine the response of S1 neurons by applying innocuous brushing and noxious pinch stimuli to the right hind paw as conventionally done in pain studies. However, since these two stimuli with the different noxiousness feature also have distinct texture and dynamics features, we added another innocuous stimulation, Press, in this first experiment session (Figure 1A, Table 1). Our idea is that if the neural response patterns to Press are similar to those to Brush, but not to Pinch, it indicates a fine-tuning of S1 neurons to the noxiousness feature; in the opposite case, it means that S1 neurons are highly tuned to the texture or dynamics feature.

To analyze neuronal response patterns to different stimuli, we calculated the preference index (PI) of individual cells to each stimulus based upon their response amplitude and fidelity (see “Materials and Methods” section). About a half of the responding neurons (fluorescence change >30% of *F*_0_) were tuned to all the three stimuli (50.2%) and the majority of the other preferentially responded to either of Brush (17.0%) or Press·Pinch (13.4%). Interestingly, Press-responsive neurons also exhibited Ca^2+^ responses to Pinch with higher amplitude, rather than to Brush (Figures 1B,C). PI scatter plots between two stimuli indicated that S1 neurons have low selectivity to Press vs. Pinch, but high selectivity to Press (or Pinch) vs. Brush (*n* = 217 cells from four mice, Figure 1D). PCA, which represents population activity patterns, also showed that Press and Pinch evoke distinct, but very close neural population responses from each other, which were clearly separated from those of Brush (*N* = 4 mice, Figures 1E,F). Taken together, these results suggest that S1 neurons are more finely tuned to the texture or dynamics feature compared to the noxiousness/intensity feature.

### Encoding Texture and Dynamics Features of Innocuous Stimuli by S1 Neurons

To comprehensively investigate how S1 neurons differentially encode the texture and the dynamics of mechanical stimuli, we recorded neuronal Ca^2+^ activity in the S1 cortex evoked by Brush-stroke (B-stroke), Brush-press (B-press), Forceps-stroke (F-stroke) and Forceps-press (F-press) hind paw stimuli (Figure 2A, Tables s 1, 2). B-stroke and F-press are relabeled terms of Brush and Press stimulus in Figure 1, respectively, and in addition, B-press and F-stroke were added for more comprehensive investigation. From a variety of response patterns of individual neurons (Figures 2A,B), we found that B-stroke (F-stroke) responsive neurons also showed Ca^2+^ activities in response to B-press (F-press), rather than to F-stroke/press (B-stroke/press, respectively). The proportion of texture-discriminative neurons, preferentially responding to B-stroke and B-press (F-stroke and F-press) regardless of the dynamics feature, was much higher than that of dynamics-discriminative neurons, preferentially responding to B-stroke and F-stroke (B-press and F-press) stimulus regardless of the texture (*n* = 208 cells from four mice, Figure 2C). Hierarchical clustering analysis suggests that S1 neurons are primarily categorized by their Ca^2+^ responses to the different textures, and secondarily by those to the different dynamics (Figure 2D). PI scatter plots also indicate that S1 neurons have relatively low selectivity to the dynamics (i.e., F-press vs. F-stroke), but show high selectivity to the texture (i.e., F-press vs. B-press; *n* = 208 cells from four mice, Figure 2E). PCA also showed that neural population response patterns to four different stimuli can be separated, but B-stroke evokes similar response patterns to those by B-press, while relatively distinct from those by F-stroke/press (Figures 2F,G). Taken together, these results suggest that S1 neurons are more selective to the texture than dynamics at individual cell level.

### Encoding Noxiousness/Intensity Features of Stimuli by S1 Neurons

Next, we sought to identify the encoding strategy of S1 neurons for the noxiousness/intensity feature of mechanical stimuli, to which S1 neurons appear to be widely tuned (to Press and Pinch; Figure 1, Press-specific, 0.92%; Pinch-specific, 5.99%; Both, 13.4%). We applied graded Forceps-pinch (F-pinch) stimuli (P0 < 2 g: noxiousness = innocuous; P1 = 100 g, P2 = 200 g and P3 = 300 g pressure: noxiousness = noxious) to the hind paw, all of which have the same texture/dynamics feature (Tables s 1, 2, Figures 3A,B). We found various response patterns of individual neurons. Interestingly, we identified “intensity coding neurons” in a certain amount of the imaged cells (Figure 3B_i_, 21.93%), which show a positive correlation of Ca^2+^ amplitude with the stimulus intensity. Some other neurons (Figure 3B_ii_, 15.30%) exhibited similar amplitudes of Ca^2+^ responses to the stimuli with four different intensities, but the neurons showing P0 (innocuous)-preference or inverse correlation of their Ca^2+^ amplitude with the stimulus intensity were rarely detected (Figure 3B_iii_, 1.53%). The remaining neurons (61.24%) showed irregular patterns of Ca^2+^ responses to the stimuli with different intensities (Figure 3B_iv_). The positive relationship between the stimulus intensity and the proportion of responding cells was observed in a non-linear fashion with steep and gentle slopes (*N* = 6 mice, Figure 3C). We also found such a non-linear positive relationship between the stimulus intensity and the average response fidelity (Figure 3D) or amplitude (Figure 3E) of S1 neurons, which are reflected in the RIs (see “Materials and Methods” section) of individual cells in response to the graded F-pinch stimuli (*n* = 197 cells from six mice, Figure 3F). These results suggest that the stronger the stimuli, the more S1 neurons are recruited, evoking stronger Ca^2+^ responses represented by higher amplitude and fidelity.

### Differential Selectivity of S1 Neurons to Multiple Stimulus Features of Brushing and Pinch

The results so far indicated that S1 neurons have different levels of selectivity for the given stimuli with different features. To more clearly determine selectivity properties of S1 neurons for multiple stimulus features of the stimuli, we reanalyzed the obtained data in Figures 1, 2 using only non-broadly tuned neurons (i.e., neurons with selectivity to specific features), except for neurons that were tuned to all types of stimuli. PI scatter plots of non-broadly tuned neurons were generated between two stimuli with only a single difference of features: noxiousness, dynamics, or texture (*n* = 101 cells from six mice, Figure 4A). It turned out that a certain amount of individual S1 neurons show a highly specific response to the difference in texture, but low specificity to the difference in dynamics or noxiousness. Between the latter two features, neurons were slightly more specific to dynamics than noxiousness. Indeed, the average z-distance between PIs and “equally tuned” lines (gray line) for pairs of stimuli were significantly positive only in the discrimination of texture, meaning the non-broadly tuned neurons tend to be exclusive in texture coding compared to corresponding null model (Figure 4B, see “Materials and Methods” section).

### Decoding Features Using the Response Patterns of the Population Activity

Finally, we tried to decode the difference between the stimuli of noxiousness, dynamics, and texture using the response patterns of the population activity, rather than individual cells. K-nearest neighbor classifier achieved perfect performance in 10-fold cross validation in all the discrimination task-difference in noxiousness, dynamics, and texture (Figure 4C, see “Materials and Methods” section). This result suggests that the information of sensory stimuli can be efficiently represented in S1 as patterns of the population, particularly in the case of low specificity to the stimuli features, such as noxiousness and dynamics.

## Discussion

S1 cortex integrates sensory information from diverse afferent sources, leading to perception of the location, intensity, quality of touch, and pain (Vierck et al., 2013). However, it remains largely unknown how the neural circuits in S1 inclusively process such various features at the single-cell and population levels. In this study, we determined how diverse features of cutaneous inputs are encoded in layer 2/3 S1 cortex of the mouse. We found that different aspects of the stimuli are encoded with different levels of selectivity at the individual neuron level. Under the stimuli conditions given here, texture was the most dominant feature that was selectively encoded at the single-cell level, followed by dynamics, and noxiousness. However, it turned out that the stimulus features with low neuronal selectivity can be successfully decoded by the supervised machine learning technique, implying the distributed information encoding of such features. Our findings suggest that S1 neurons encode multiple stimulus features of touch and pain at the individual cell and population levels in a feature-dependent manner.

Previous electrophysiological studies characterizing S1 neurons for noxiousness in animals demonstrated that the proportion of nociceptive specific HT neurons is much smaller than that of non-nociceptive LT and convergent WDR neurons (Lamour et al., 1983; Kenshalo et al., 2000). Our results also showed that a majority of S1 neurons responded to non-nociceptive brushing/press stimuli and exhibited highly selective responses toward non-nociceptive texture features derived from a brush or a forceps steel arm. Previous *in vivo* studies of S1 barrel cortex have reported that layer 2/3 neurons show preferred response patterns to specific texture coarsenesses, while a minority of neurons respond monotonically to the graded texture coarsenesses (Garion et al., 2014; Bourgeon et al., 2016). Taken together, these results imply that texture features of tactile information are well discriminated at the individual cell level in S1.

Traditionally, cutaneous sensory information is thought to be conveyed from peripheries to the cortex *via* independent neural pathways according to their submodality, which is characterized by response properties of afferent classes: rapidly adapting (RA), slowly adapting type 1 (SA1), slowly adapting type 2 (SA2), and pacinian (PC) afferents. These led to the notion that neurons in relatively high levels of the sensory system such as the thalamus and S1 are highly selective to specific submodality as in the periphery. Recent evidence, however, shows that individual neurons in S1 receive inputs from multiple afferent classes, and therefore should not be defined based on submodality, but on their function (Saal and Bensmaia, 2014). Our results are consistent with this emerging evidence; about half of the analyzed neurons responded to both static and dynamic stimuli, with the former indicating SA 1 input, the latter RA or PC input. These results evidently indicate the submodality convergence rather than its segregation in the S1 cortex during touch sensation. More importantly, we revealed that S1 neurons are primarily tuned to texture features, rather than the noxiousness or intensity feature. This raises concerns about using the conventional concept of LT/HT/WDR in classifying cortical or thalamic neurons in pain studies. Indeed, it was already reported that thalamic neurons are not adequately classified by this classification scheme. Clustering results of thalamic neurons based on the response properties to several tested stimuli were different from that of spinal cord neurons (Chung et al., 1986). Using classification scheme of LT/HT/WDR in the brain is based on the assumption that relatively well-defined concept in the spinal cord will be preserved at a higher level by segregated channels, however, our data and the previous evidence suggest that the modality of noxiousness is intermixed in S1, as well as submodalities of touch sensation. Therefore, it is necessary to develop an objective method to describe S1 neurons by their function (response properties) based on quantitative data rather than predefined modality such as LT, HT or “nociceptive-specific.”

Our study also examined how different pain intensities are represented in the S1 neurons. We observed response of S1 neurons to the noxiousness/intensity feature by applying graded F-pinch stimuli with the same Texture/Dynamics feature but only with different intensities. Most of the neurons exhibited irregular or broadly tuned responses to the graded F-pinch stimuli. At the population level, however, we found that more S1 neurons are recruited and stronger Ca^2+^ responses are evoked as the stimulus intensity is increased in a nonlinear manner. This result agrees with the previous studies showing that the stimulation intensity is positively correlated with S1 neuronal responses in a nonlinear fashion (Timmermann et al., 2001; Bornhövd et al., 2002; Eto et al., 2011). It also should be noted that a subset of S1 neurons show gradually increased amplitude of Ca^2+^ transients with increasing stimulus intensity and exhibit a linear relationship for stimulus intensity. It would be challenging, but of high interest, to further clarify the distinct functional and genetic properties of these “intensity coding neurons” in the future study. Nevertheless, these neurons could be potentially used for the objective measurement of the degree of pain or the efficacy of analgesics.

In our study, a majority of S1 neurons responded to more than two types of stimuli, rather than selectively responding to each texture, dynamics or noxiousness, indicating that individual S1 neurons encode multiple features of sensory information. Given the multifaceted nature of the sensory information in real life setting, this is a reasonable and efficient strategy to process numerous types of distinct stimuli within a limited sensory system resource (Chu et al., 2016). Indeed, similar phenomena have been reported in complex cognitive tasks of the prefrontal cortex, known as “mixed selectivity” (Rigotti et al., 2013; Ramirez-Cardenas and Viswanathan, 2016; Parthasarathy et al., 2017). Thus, our findings extend this “mixed selectivity” concept to the somatosensory cortex, suggesting that it is a more general mechanism in the cortex than previously thought.

All the data in this study were obtained from layer 2/3. Neurons in layer 2/3 receive inputs mainly from layer 4, which is a target layer for thalamic projections to S1, and predominantly project to layer 5. There are also abundant local connections within layer 2/3 (Lefort et al., 2009). Previous *in vivo* electrophysiological studies in barrel cortex have demonstrated that whisker stimulation evokes subthreshold depolarization of layer 2/3 neurons with much broader receptive fields than in layer 4 (Brecht and Sakmann, 2002; Brecht et al., 2003; Kim T. et al., 2016). These results suggest that somatotopically organized input from the thalamus along layer 4 becomes intermingled in layer 2/3. Related to these results, our findings of differential selectivity for different aspects of the sensory information in layer 2/3 might provide a cross-sectional view of the complex sensory information processing for multiple features beyond receptive fields. It will be interesting to compare the selectivity of the neurons for features we analyzed in this study in upstream areas such as layer 4 and the thalamus.

It is known that about 80% of the neocortical neurons are excitatory pyramidal neurons and the remaining 20% are GABAergic inhibitory neurons (Markram et al., 2004). Recorded neurons in our study may be comprised of the same proportion of excitatory and inhibitory neurons if the sampling was not systematically biased. However, we do not perfectly rule out the possibility of sampling bias in our study. For example, neurons with low burst activity in the anesthetized state could have less chance of the selection for analysis, and this might introduce some difference in the proportion of excitatory and inhibitory neurons in our data. There is evidence that most excitatory and inhibitory neurons in layer 2/3 of S1 cortex in awake mice increase their action potentials during passive and active whisker deflection except for somatostatin-expressing neurons, which reduce the tonic firing rates in response to whisker sensing (Gentet et al., 2012). However, there were no neurons showing decreased calcium response to stimuli in our experiments, and this is presumably because such neurons could not be selected for analysis due to their low calcium activity under anesthesia. Future experiments investigating cell type-specific recordings in awake mice are needed to gain a more detailed understanding of the encoding mechanism of S1 circuits for diverse features of sensory information.

Some limitations of our study are worth mentioning: (1) all the experiments in this study were performed under isoflurane anesthesia. The anesthesia was inevitable since it is extremely difficult to repeatedly stimulate the same regions in awake animals and to control other sensory inputs from movements. It has been shown that the anesthesia reduces the tuning properties of neurons to stimuli in the V1 and A1 cortex of rodents (Gaese and Ostwald, 2001; Goltstein et al., 2015). Thus, we cannot completely rule out the possibility that the evoked responses of the neurons are influenced by the isoflurane anesthesia, although it is unlikely that such changes will appear in a feature-dependent manner; (2) there exist the ambiguity in the comparison design of multiple stimulus features in our study. For example, the difference of the qualitative texture between brush and forceps steel arm cannot be directly comparable to the intensity difference of quantitative pinch stimuli, such as 100 g and 300 g. Furthermore, there might be confounding features that could bias our interpretation of the selective response of neurons, such as temperature or indentation depth of the stimuli. In other words, selective responses of neurons for the different texture stimuli might be caused by the subtle difference in surface temperature or intensity of pressures between the brush and the forceps steel arm. To rule out this possibility, first, we measured the surface temperature of brush and forceps using an infrared thermometer (Supplementary Figure S1A). The temperature difference between the two stimulation tools was only 0.5°C. This tiny difference does not cause selective responses of S1 neurons (Milenkovic et al., 2014). We then applied two pressures with different intensity (20 g and 50 g) to the hind paw in a random order, assuming that the difference in indentation depth induced by the two stimulation tools is not as large as those by these two pressure stimuli (*N* = 3 mice, Supplementary Figures S1B–D). PI scatter plots showed that the neurons have similar response patterns to the pressure stimuli with different indentation depth in terms of the fidelity and response amplitude. Calcium response amplitude of cells differed between the two pressures, but the proportion of the responding cells was not significantly different. Although there are several neurons with a difference in response amplitudes between the pressures, it is difficult to say that the small difference in force has contributed to the selective response in S1 individual neurons (Ferrington et al., 1988; Moehring et al., 2018). Therefore, it is unlikely that subtle differences in temperature or indentation depth caused by the stimuli with the brush and forceps affect the observed results in this study; (3) it is worthy to note that there are differences of the organization, gene expression patterns, and the response characteristics of cortical neurons between species (Kenshalo et al., 2000; Hutsler et al., 2005; Zeng et al., 2012). For example, there have been some discussions about the distinct response properties to sensory stimuli of the different cytoarchitectural areas of S1 (such as 3b, 3a, 1, and 2; Vierck et al., 2013), yet our results cannot suggest valuable insights with regard to these issues since these cytoarchitectonic distinction does not exist in mice. The fact that layers 2 and 3 are greatly expanded layers in the cortex of primates compared to rodents might cause bias in translating our results to humans. It would be a valuable work to verify our results in primates by applying the experimental design and analytical approaches employed in this study.

In conclusion, we demonstrated the differential selectivity of S1 neurons for multiple stimulus features of brushing and pinch. The majority of tuned neurons selectively responded to texture features rather than noxiousness features, implying that conventional classification of neurons (LT, HT, and WDR) in pain studies cannot be simply employed in the S1 cortex. Sensory stimuli could be decoded *via* patterns of neural population activity, even for the features with low specificity at the individual cell level. We also showed a group of neurons in the S1 cortex encodes pain intensity by amplitude and fidelity. Our results provide an important insight into the encoding strategy of S1 neural circuits for multiple stimulus features of touch and pain.

## Ethics Statement

All experimental procedures were approved by the Seoul National University Institutional Animal Care and Use Committee and performed in accordance with the guidelines of the National Institutes of Health.

## Author Contributions

YK, SKK and SJK conceived and designed the study. YK and HY performed the experiments. C-EK developed analytic tools and MATLAB codes. YK and C-EK analyzed the data. SKK and SJK supervised the experiments and analyses. YK, C-EK, SKK and SJK wrote the manuscript. All of the authors read and discussed the manuscript.

## Conflict of Interest Statement

The authors declare that the research was conducted in the absence of any commercial or financial relationships that could be construed as a potential conflict of interest.
