# Supplementary material for: S1 Employs Feature-Dependent Differential Selectivity of Single Cells and Distributed Patterns of Populations to Encode Mechanosensations
Source: Front Cell Neurosci. 2019 Apr 5;13:132. doi: 10.3389/fncel.2019.00132 (PMC6460949; doi:10.3389/fncel.2019.00132)
Supplement: Supplementary file 1 [file Image_1.pdf]

frontiers

Supplementary Material

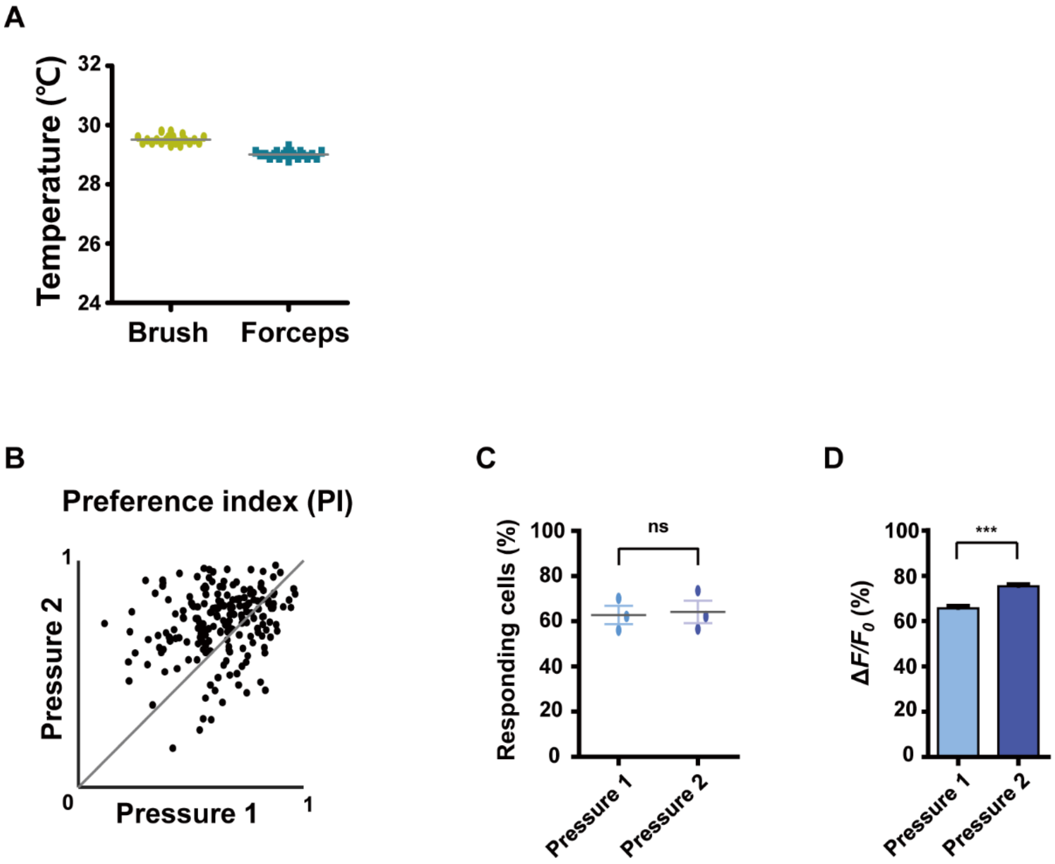

Supplementary Figure 1 | Effects of the temperature and indentation depth of the skin caused by the stimuli with the brush and forceps. **(A)** Surface temperature of brush and forceps measured by an infrared thermometer (Brush, 29.51℃ ± 0.14℃; forceps, 29.01℃ ± 0.12℃). Each stimulation tool was repeatedly measured 20 times. **(B)** Scatter plots of the preference indexes (PIs) of individual neurons for two different pressure: Pressure 1 versus Pressure 2 (n = 191 cells from 3 mice). Pressure 1 and 2 corresponds to 20g and 50g, respectively. **(C)** The relationship between the number of responding cells and the stimulus pressure (N = 3 mice; Wilcoxon signed rank test, ns). **(D)** The relationship between Ca^{2+} transients amplitude and the stimulus pressure (n = 191 cells from 3 mice; Two-tailed paired t-test, $p < 0.0001$). All data are represented as mean ± s.e.m. NS indicates $p > 0.05$ and *** indicates $p < 0.001$.
